# Case Report: Successful Avatrombopag Treatment for Two Cases of Anti-PD-1 Antibody-Induced Acquired Amegakaryocytic Thrombocytopenia

**DOI:** 10.3389/fphar.2021.795884

**Published:** 2022-01-27

**Authors:** Xiaofang Tu, Ali Xue, Suye Wu, Mengmeng Jin, Pu Zhao, Hao Zhang

**Affiliations:** Department of Hematology, The Third Affiliated Hospital of Wenzhou Medical University, Wenzhou, China

**Keywords:** immune-related adverse events, acquired amegakaryocytic thrombocytopenia, anti-PD-1 antibody, thrombopoietin receptor agonists, avatrombopag

## Abstract

**Background:** Anti-PD-1/PD-L1 immunotherapy has achieved impressive responses in multiple types of malignancies in recent years. However, immune-related adverse events (irAEs) occur and limit their continuous clinical use. Among these irAEs, acquired amegakaryocytic thrombocytopenia (AAT) is rare but often clinically serious, life-threatening and refractory to multiple treatment approaches.

**Case summary:** We reported for the first time the successful treatment of avatrombopag in two cases of anti-PD1 antibody-induced AAT (in particular, one case had progressed to aplastic anemia), which was refractory or intolerant to glucocorticoids, ciclosporin, intravenous immunoglobulin (IVIG), recombinant human thrombopoietin (rh-TPO) and even TPO receptor agonist (TPO-RA) eltrombopag. To date, the two cases manifested as normal platelet counts and are independent of transfusion.

**Conclusion:** Anti-PD1 antibody-induced AAT occurs with low frequency but is often serious and difficult to manage, for which this study proposed vatrombopag as a potential curative and safe approach.

## Introduction

Programmed cell death protein 1 (PD-1) is a member of the immunoglobulin supergene family that is expressed upon lymphocyte activation in CD4^+^ and CD8^+^ T cells, which acts as a natural brake that modulates the T cell response. Blockade of the PD-1/programmed cell death ligand 1 (PD-L1) pathway by monoclonal antibodies has emerged as a highly effective approach to reinvigorate T cells in treating several types of malignancies such as melanoma, lung cancer, renal cell carcinoma, gastric cancer and certain types of lymphoma. However, immune-related adverse events (irAEs) frequently occur and can potentially affect all organs, which limits the continued use of anti-PD1/PD-L1 antibodies (De Velasco et al., 2017).

Hematological irAEs induced by anti-PD-1/PD-L1 immunotherapy are much less frequent than those induced with conventional cytotoxic chemotherapy and account for approximately 3.6% of total irAEs with the most common type of neutropenia, autoimmune hemolytic anemia and immune thrombocytopenia each in 26%, followed by pancytopenia or aplastic anemia in 14% (Delanoy et al., 2019; Michot et al., 2019). In particular, a rare hematological disorder, which is called acquired amegakaryocytic thrombocytopenia (AAT), is characterized by severe thrombocytopenia and a complete or nearly complete absence of megakaryocytes in the bone marrow (Agarwal et al., 2006). AAT is distinguished from megakaryocyte maturation disorder in immune thrombocytopenia (ITP). AAT is often clinically serious and life-threatening due to the significantly increased risk of vital organ bleeding. To date, only sporadic cases of anti-PD-1/PD-L1 immunotherapy-induced AAT have been reported. The standard treatment of AAT has not been defined, and the management is often thorny because of their refractoriness to possible treatment choices, including immunosuppressive therapy, rituximab, interleukin-11, recombinant human thrombopoietin (rh-TPO) and even some thrombopoietin receptor agonists (TPO-RAs). As a newly FDA approved TPO-RA for immune ITP, avatrombopag promotes platelet production by stimulating TPO receptor (c-Mpl) with high efficacy and safety (Deng et al., 2021; Gilreath et al., 2021). However, little is known about its effects in the treatment of AAT, especially anti-PD-1/PD-L1 antibody-induced AAT. In this study, we report for the first time the successful treatment of avatrombopag in two patients with anti-PD-1 antibody-induced AAT.

## Case Presentations

### Patient 1

A 67-year-old male was diagnosed with ureter neoplasm with right hydronephrosis and retroperitoneal lymph node metastasis in May 2020. After three cycles of combined chemotherapy (gemcitabine and carboplatin), the patient was administered tislelizumab at a dose of 200 mg every 3 weeks. Three weeks after the second treatment with tislelizumab, routine blood examination indicated thrombocytopenia with a platelet count of 4.8×10^4^/µL. Tislelizumab was discontinued, and rh-TPO was used at a dose of 15,000 U/day for 13 consecutive days, but repeated examination showed a further decreased platelet count of 2.1×10^4^/µL. Considering anti-PD-1 antibody-related immune thrombocytopenia, the patient received methylprednisolone 80 mg daily for three consecutive weeks until skin hemorrhages and petechiae appeared on his extremities and abdomen, and the platelet count decreased to 0.5×10^4^/µL. Bone marrow morphology showed an almost absence of megakaryocytes with no significant abnormal presentation of other cell linages. Excluding other possible secondary thrombocytopenia (other immune diseases, drugs, or infections induced thrombocytopenia), anti-PD-1 antibody-induced AAT was considered. Due to the risk of life-threatening bleeding, the patient received intravenous immunoglobulin (IVIG) 20 g/day for 5 days and irregular platelet infusion. Unfortunately, there was still no improvement in his platelet count. The following administration of cyclosporine 100 mg daily was discontinued 1 week later because severe pneumonia occurred.

**FIGURE 1:**
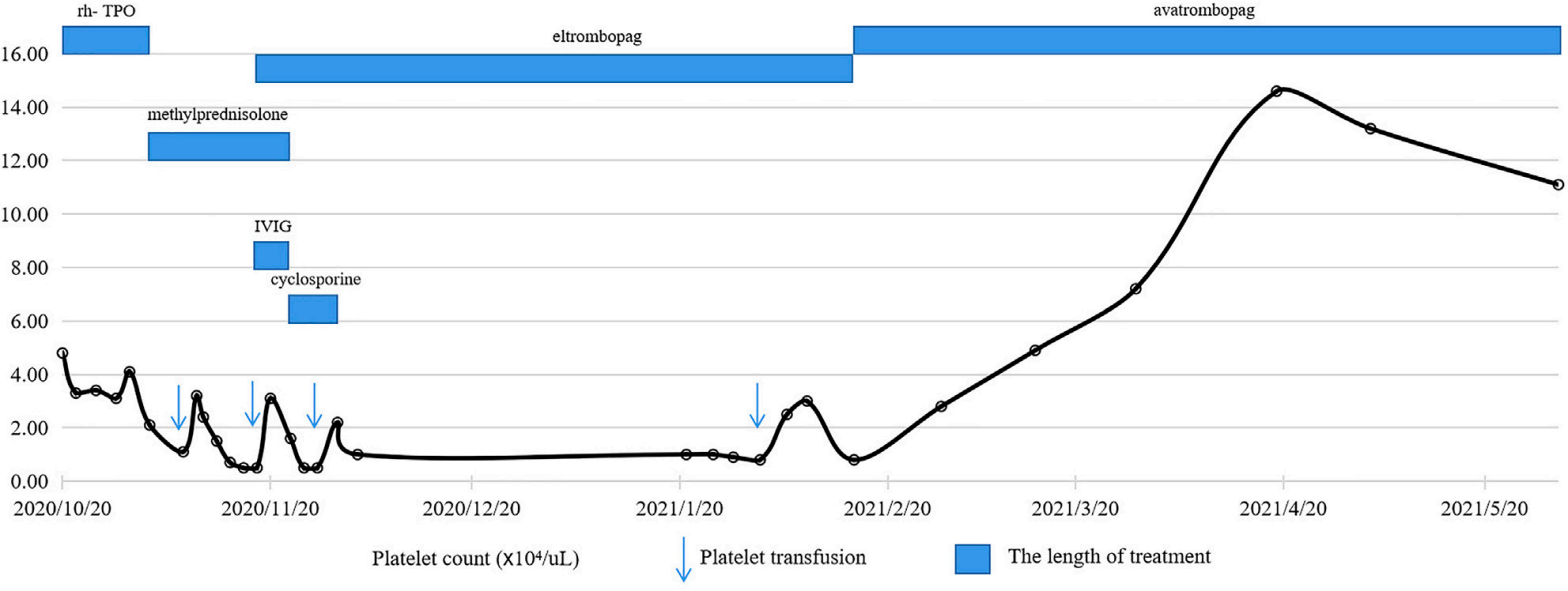
Schedule of treatment and dynamics of platelet counts for patient 1. After two courses of tislelizumab treatment, the platelet count gradually decreased with the lowest level of 0.5×10^4^/µL, which was refractory to glucocorticoids, IVIG, rh-TPO, cyclosporine and eltrombopag. With the administration of avatrombopag 40 mg daily, the platelet count rapidly increased and remained above 10×10^4^/µL.

As a potentially effective strategy, TPO-RAs were considered. Oral eltrombopag 50 mg daily was initiated and lasted for a total of 3 months, but it ended in limited responses. The patient was transitioned to avatrombopag at a dose of 40 mg daily. Surprisingly, the platelet count increased to 2.8×10^4^/µL 2 weeks later and above 10×10^4^/µL 2 months after the avatrombopag initiation. To date, the platelet count of the patient has remained normal (Figure 1), and no obvious adverse effects have been observed.

### Patient 2

A 71-year-old female who was diagnosed with bladder cancer received surgical resection of the tumor and four cycles of chemotherapy (gemcitabine and cisplatin) in 2019. Due to the evaluation as a poor prognosis, she was started on treatment with triplezumab 200 mg every 3 weeks. After two courses of treatment, she developed thrombocytopenia with a platelet count of 2.6×10^4^/µL. Rh-TPO was given at a dose of 15,000 U daily for 17 consecutive days, and the platelet count returned to 5×10^4^/µL. Approximately 1 month later, repeated routine blood examination showed a decreased platelet count of 2.9×10^4^/µL. Bone marrow examination demonstrated megakaryophthisis, and AAT was diagnosed in the absence of evidence that other causes induced thrombocytopenia. Five days of IVIG (20 g/day) and 2 weeks of rh-TPO (15,000 U/day) were administered, but no response was observed, and the platelet count continuously decreased to 1.5×10^4^/µL. Then, she received eltrombopag 50 mg daily but discontinued because of economic considerations and limited responses within 2 weeks.

At 5 months later, the patient developed bleeding on her skin and gums with severe thrombocytopenia (platelet count 0.2×10^4^/µL), anemia (hemoglobin 5.7 g/dl) and granulocytopenia (white blood cell count 1.9×10^3^/µL). Repeated bone marrow examination suggested multilineage hypoplasia with a near absence of megakaryocytes, and immune-related aplastic anemia was diagnosed. The combination of methylprednisolone (40 mg/day), rh-TPO (15,000 U/day) and granulocyte colony-stimulating factors (G-CSF) (200 U/day) was administered for 2 weeks, and no response was observed. Considering the age and potential immunocompromise, the patient refused the use of cyclosporin.

**FIGURE 2:**
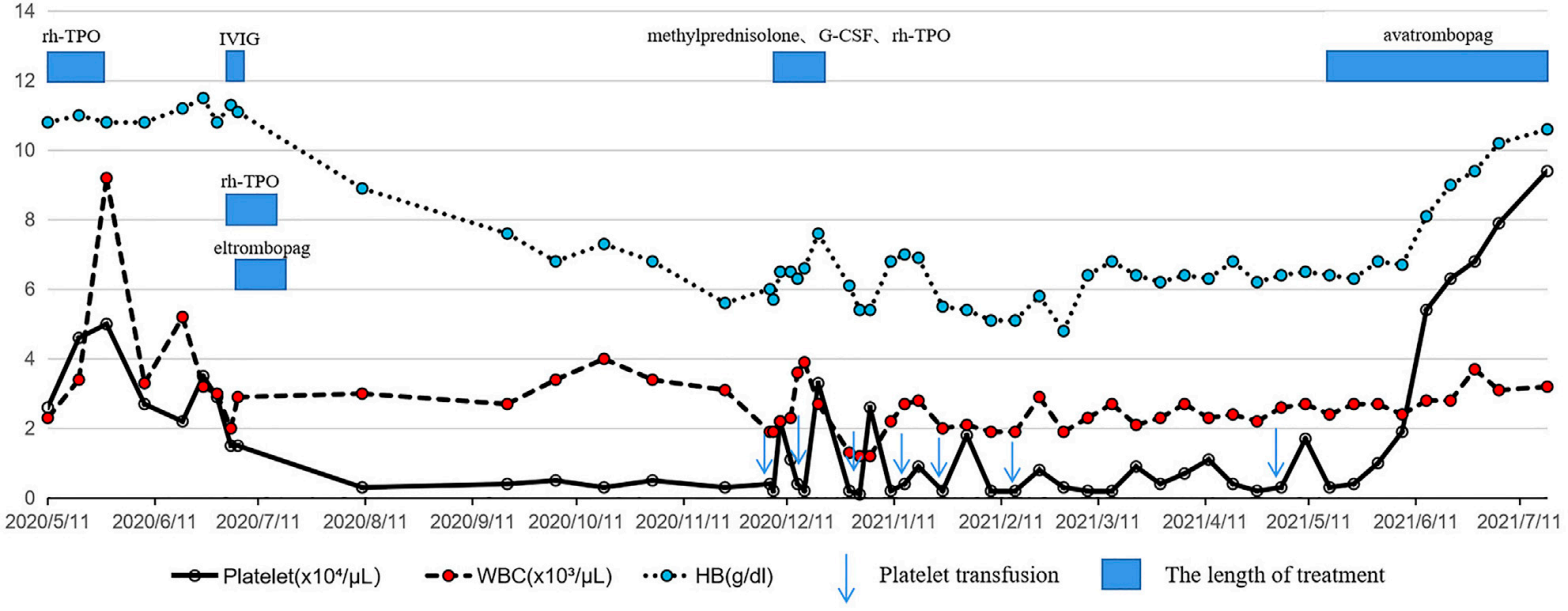
Schedule of treatment and the dynamics of white blood cells, hemoglobin and platelets for patient 2. Two courses of triplezumab treatment induced a gradually decreased platelet count followed by pancytopenia with no response to glucocorticoids, IVIG, rh-TPO or eltrombopag. With the use of avatrombopag 40 mg daily, a rapid increase in platelet count, hemoglobin and white blood cell count was observed and maintained.

After another 5 months, the patient agreed to the treatment of avatrombopag because of sustained pancytopenia and accompanying complications. Encouragingly, at a dose of 40 mg daily for 1 month, routine blood examination showed a significant improvement in blood cell count. Two months after the avatrombopag administration, a satisfactory blood cell count was achieved with a platelet count of 9.4×10^4^/µL, a hemoglobin count of 10.6 g/dl and a white blood cell count of 3.2×10^3^/µL (Figure 2). During follow-up, no significant adverse events were observed with continued use of avatrombopag.

## Discussion

With the widespread application of anti-PD-1/PD-L1 antibodies in multiple malignancies, irAEs have been frequently observed in the clinic. Hematological irAEs, especially AAT, occur with a low frequency, but severe bleeding is often life-threatening. Although immune-related disorders are thought to be causative, the exact underlying pathogenesis remains unclear. The possible mechanisms associated with AAT involve activated T cells (T suppressor activated lymphocytes (Benedetti et al., 1994), T-cell large granular lymphocytes (Rajpurkar et al., 2019)), humoral immunity (anti-TPO antibody (Shiozaki et al., 2000), anti-c-Mpl antibody (Son et al., 2019)) or cytokine (Kimura et al., 1996)-mediated disorders of megakaryocyte generation. For anti-PD-1/PD-L1-induced AAT specifically, the underlying mechanism may be similar to those antitumor responses, which refer to the expansion of the T-cell repertoire. Iyama S et al. suggested that cell-mediated instead of humoral immunity possibly participated in anti-PD-1 antibody-induced AAT in studies where no significant difference was observed in growth effects between patient and control plasma samples on BFU-E, CFU-GM and CFU-GEMM (Iyama et al., 2020). The role of affected B cell responses and induced autoantibody production, such as anti-TPO, anti-c-Mpl or platelet-associated IgG, in anti-PD-1/PD-L1-induced AAT is unclear due to the limited number of cases and the absence of related clinical and experimental data.

Published reports demonstrated preserved megakaryocytes in most cases of anti-PD-1/PD-L1-induced immune thrombocytopenia, some of which were successfully treated with glucocorticoids, cyclosporins (IVIGs), rituximab or TPO-RA (Suyama et al., 2021). However, based on the possible different pathogenesis in anti-PD-1/PD-L1-induced AAT, treatment responses greatly varied in limited individuals. Thus, effective or optimal therapeutic choices remain confusing and controversial. Nishino S et al. (Nishino et al., 2018) reported a case with anti-PD-1/PD-L1-induced AAT that responded dramatically to glucocorticoids, while the case provided by Suyama T et al. did not respond to glucocorticoids but was cured by TPO-RA eltrombopag (Suyama et al., 2021). Nevertheless, in the present two cases of this study, tislelizumab/triplezumab-induced AATs were refractory to glucocorticoids, IVIG, rh-TPO and even TPO-RA eltrombopag. In addition, due to the potential risk of infection or tumor progression caused by immunocompromise in patients with malignancies, the application of long-term or strong immunosuppressive therapy was restricted, such as cyclosporin and anti-thymocyte globulin.

Similar to eltrombopag, avatrombopag specifically binds to the TPO receptor (c-Mpl) and stimulates the proliferation and differentiation of megakaryocytes and even hematopoietic stem cells (HSCs), which results in resumed production of platelets. Due to its safety and effectiveness, avatrombopag was approved by the FDA to treat chronic ITP in adults (Provan et al., 2019) and thrombocytopenia in adult patients with chronic liver disease (Shirley, 2018). According to the manufacturer’s instructions, a daily dose of 20–40 mg is recommended for patients with chronic ITP, and the standard length of treatment is not defined. TPO-RAs differ in their specific molecular structure, pharmacokinetic characteristics, binding site and the manner in which they stimulate the TPO receptor. Thus, patients who experience intolerance or lack of efficacy with one TPO-RA may benefit from switching to an alternate TPO-RA (Khellaf et al., 2013; Lakhwani et al., 2017). Surprisingly, by transitioning to avatrombopag, both patients in this study achieved satisfactory hematologic responses. The different responses induced by eltrombopag and avatrombopag in the present cases could also be dose-related, since it has been reported that daily 20 mg doses of avatrombopag produce 3–5 times higher peak platelet counts than daily 75 mg doses of eltrombopag (Al-Samkari and Kuter, 2018). Meanwhile, it is also worth considering whether the duration of treatment affects the outcome, since the length of eltrombopag administration was only 2 weeks, and avatrombopag lasted several months in patient 2. No obvious adverse reactions were observed with continued use of avatrombopag in the two cases, although pooled data from clinical trials in ITP indicated that some patients experienced headache, fatigue, epistaxis, infections, etc.

Of note, because anti-PD-1/PD-L1 antibody-induced immune attack can theoretically affect all types of cells, including HSCs and myeloid progenitors, some AATs may progress to aplastic anemia (Delanoy et al., 2019; Novotny et al., 2017). Based on TPO receptors expressed on HSCs, TPO-RA treatment was associated with multilineage clinical responses in some patients with aplastic anemia (Olnes et al., 2012; Scheinberg, 2018). To our knowledge, the application of avatrombopag in aplastic anemia, particularly anti-PD-1/PD-L1 antibody-induced aplastic anemia, has not been reported. The nearly complete response achieved by avatrombopag in patient 2 with AAT progression into aplastic anemia proposed a promising therapeutic approach for such diseases.

In conclusion, anti-PD-1/PD-L1 antibody-induced AAT is rare but often serious and refractory to multiple treatments. This study proposed avatrombopag as a potential curative and safe approach for such patients with limited efficacy or intolerance to immunosuppressive therapy or other recommended treatments.

## Data Availability

The original contributions presented in the study are included in the article/supplementary material, further inquiries can be directed to the corresponding authors.

## Abbreviations

PD-1, programmed cell death protein 1; PD-L1, programmed cell death ligand 1; irAEs, immune-related adverse events; AAT, acquired amegakaryocytic thrombocytopenia; ITP, immune thrombocytopenia; rh-TPO, recombinant human thrombopoietin; TPO-RAs, thrombopoietin receptor agonists; IVIG, intravenous immunoglobulin; G-CSF, granulocyte colony-stimulating factors; HSCs, hematopoietic stem cells
